# Development of an Integrated Electronic Platform for ADHD Medication Initiation in Child and Adolescent Mental Health Services

**DOI:** 10.1192/bjo.2024.90

**Published:** 2024-08-01

**Authors:** Noah Stanton, Hamzah Iqbal, Sandra Bailey, Louise Morganstein, Salim Jakhra

**Affiliations:** Central & North West London NHS Foundation Trust, London, United Kingdom

## Abstract

**Aims:**

Children and young people (CYP) with attention deficit hyperactive disorder (ADHD) under Brent Child and Adolescent Mental Health Services (CAMHS) experience long waiting times before treatment with medication is initiated: on average 3 months. Therefore, the aims were firstly, to create an electronic platform (e-platform) to educate parents about ADHD medication and facilitate its initiation in Brent CAMHS. The e-platform replaced the previous method of medication initiation which was typically delivered to a group over Zoom. Secondly, to reduce time-to-treatment initiation (TTI) by January 2024. Thirdly, to increase the proportion of patients with ADHD who were initiated on medication (when appropriate) by the same date.

**Methods:**

The content from neurodevelopmental clinicians counselling parents about ADHD medication was transcribed. Individual transcripts were collated into a master transcript to standardise the information delivered to parents. Medication initiation psychoeducation videos were created using the master transcript and a videographer and editor, in collaboration with the Trust's Director of Communications and Web Development Team. The videos were integrated electronically with a question-and-answer section, a useful websites section and a medication decision section to construct an e-platform, which was embedded in the Brent CAMHS website.

Following the QI model-of-improvement, objective clinical measures included TTI, the proportion of CYP initiated on medication, and total clinical and administrative time saved. User-reported outcomes were measured using a pre- and post-intervention questionnaire combining Likert scale and free-response items.

**Results:**

TTI reduced by 37% from 92 days (Zoom) to 58 days (e-platform). The proportion of CYP initiated on medication increased from 64% (Zoom) to 72% (e-platform). Over a 2-month period, 9 hours of clinician time was saved. Based on 20 respondents, overall user satisfaction increased from 4.13/5 (Zoom) to 4.71/5 (e-platform). Qualitative feedback revealed that users found the e-platform ‘easy to understand’, ‘easy to access, quick and useful’ and ‘provided clear explanations’.

**Conclusion:**

The results indicate the positive impact of the e-platform initiative which can be derived from both clinical and user-reported outcomes. By integrating standardized educational content, user-friendly features and streamlined processes, the e-platform empowers parents with knowledge, enhances communication between families and the neurodevelopmental team, and ultimately expedites ADHD medication initiation and saves clinical time. Regional spread has commenced, and the authors are engaged in discussions with other CAMHS to facilitate this further.

